# Developing Novel Host-Based Therapies Targeting Microbicidal Responses in Macrophages and Neutrophils to Combat Bacterial Antimicrobial Resistance

**DOI:** 10.3389/fimmu.2020.00786

**Published:** 2020-06-05

**Authors:** Katie Watson, Clark D. Russell, J. Kenneth Baillie, Kev Dhaliwal, J. Ross Fitzgerald, Timothy J. Mitchell, A. John Simpson, Stephen A. Renshaw, David H. Dockrell

**Affiliations:** ^1^Department of Infection Medicine, University of Edinburgh, Edinburgh, United Kingdom; ^2^Centre for Inflammation Research, University of Edinburgh, Edinburgh, United Kingdom; ^3^Roslin Institute, University of Edinburgh, Edinburgh, United Kingdom; ^4^Institute of Microbiology and Infection, University of Birmingham, Birmingham, United Kingdom; ^5^Institute of Cellular Medicine, Newcastle University and Newcastle Hospitals NHS Foundation Trust, Newcastle upon Tyne, United Kingdom; ^6^Department of Infection, Immunity and Cardiovascular Disease, University of Sheffield Medical School, Sheffield, United Kingdom

**Keywords:** antimicrobial resistance, macrophage, neutrophil, host-based therapies, innate immunity

## Abstract

Antimicrobial therapy has provided the main component of chemotherapy against bacterial pathogens. The effectiveness of this strategy has, however, been increasingly challenged by the emergence of antimicrobial resistance which now threatens the sustained utility of this approach. Humans and animals are constantly exposed to bacteria and have developed effective strategies to control pathogens involving innate and adaptive immune responses. Impaired pathogen handling by the innate immune system is a key determinant of susceptibility to bacterial infection. However, the essential components of this response, specifically those which are amenable to re-calibration to improve host defense, remain elusive despite extensive research. We provide a mini-review focusing on therapeutic targeting of microbicidal responses in macrophages and neutrophils to de-stress reliance on antimicrobial therapy. We highlight pre-clinical and clinical data pointing toward potential targets and therapies. We suggest that developing focused host-directed therapeutic strategies to enhance “pauci-inflammatory” microbial killing in myeloid phagocytes that maximizes pathogen clearance while minimizing the harmful consequences of the inflammatory response merits particular attention. We also suggest the importance of One Health approaches in developing host-based approaches through model development and comparative medicine in informing our understanding of how to deliver this strategy.

## Introduction

Antimicrobial chemotherapy has formed the cornerstone of our therapeutic strategy against bacterial disease since penicillin was first developed. Prior to this, developing host-based therapy was a major focus, including Fleming's original work on lysozyme, a humoral microbicide he isolated while seeking antimicrobial factors in pus (1). The first therapeutic use of penicillin in 1930 (treating eye infections in babies in Sheffield by Cecil Paine), and the pioneering work of Florey, Chain and colleagues in Oxford who developed innovations in penicillin synthesis to allow the first clinical trials in 1941, established antimicrobial chemotherapy as the pre-eminent therapeutic approach to bacterial disease (2). This has had a major impact on human health but arguably diverted focus away from host-based approaches other than vaccination.

Recent public health estimates suggest antimicrobial resistant bacteria cause 131 infections/100,000 population in Europe and that two-thirds are nosocomial (3). The disability adjusted life years of these infections approximates tuberculosis, influenza and HIV combined (3). In addition, development of new antimicrobials has been declining (4). There is thus a pressing need to develop new antimicrobials, improved antimicrobial stewardship, better diagnostics to identify the patients who truly need antimicrobials, and alternative approaches, for example those involving bacteriophage therapy, nanoparticle-based therapy, photodynamic light therapy and antimicrobial peptides (AMP) to manage infection with antimicrobial resistant ESKAPE (*Enterococcus faecium, Staphylococcus aureus, Klebsiella pneumoniae, Acinetobacter baumannii, Pseudomonas aeruginosa*, and *Enterobacter* spp.) pathogens (5). While vaccination remains a major focus, the concept of developing host-based therapy is gaining traction.

## Characteristics of Optimal Innate Immune Responses to Pathogenic Bacteria

Pathogenic bacteria commonly colonize healthy individuals without causing disease. *S. aureus* is carried by >40% of infants after birth and ~50% of adults are permanent or intermittent carriers (6, 7). Uropathogenic *Escherichia coli* is typically part of an individual's fecal microbiota and healthy individuals carry a large number of potentially pathogenic strains (8). In other cases, pathogens are harmless microbiome constituents but cause opportunistic infections in patients whose immune system is impaired by medical co-morbidity, such as nosocomial enterococcal infections (9). This apparent paradox, between common carriage but uncommon disease, suggests most infections are readily controlled by the host yet the specific microbicidal responses that control infection when small numbers of colonizing bacteria translocate to new sites is incompletely defined. Broadly, the innate immune system ensures a rapid response, working in concert with any adaptive immune responses to the pathogen. There are many components to the innate immune system including mucosal barrier function, humoral factors released in mucosal secretions and a range of innate cellular responses that are not restricted to myeloid phagocytes but also include innate lymphoid cells. These responses are modified through adaptive immune responses, but the focus of this review is exclusively on myeloid phagocyte responses.

Professional phagocytes (macrophages and neutrophils) clear bacteria from mucosa associated with a low-density microbiome, for example the distal airway or bladder (10). Macrophages play a critical role in the initial response as the resident phagocytes in tissues, using pattern recognition receptors (PRRs) to detect pathogens and orchestrate the inflammatory response. They are efficient at phagocytosing bacteria and utilize a range of microbicidal strategies to kill ingested bacteria. Tissue macrophage function is tightly controlled by activation state which is regulated by a cell network including epithelial, endothelial, T- and B- lymphocytes, as well as tissue resident innate lymphoid cells. The resulting cytokine networks reflect the importance of environmental cues (11). Innate immune memory ensures previous pathogen exposure modulates macrophage function via epigenetic imprinting of monocytes to induce “training” (enhanced microbicidal responses to repeat challenge) and “tolerance” (reduced deleterious responses to repeat challenge) to pathogen-associated molecular patterns (12, 13). Lipopolysaccharide (LPS) engagement of Toll-like receptor (TLR) 4 is just one example amongst several of a microbial stimulus that can on repeat stimulation be associated with tolerance manifest as reduced generation of pro-inflammatory cytokines and reactive species (14). This has implications for monocyte-derived macrophage populations but the extent to which it also influences resident macrophage populations with distinct ontogeny remains to be established. Though capable of avid phagocytosis, tissue macrophages have a finite capacity to kill ingested bacteria (15). This capacity can be diminished by interactions with other microorganisms e.g., viruses, environmental factors or co-morbidity, resulting in increased susceptibility to bacterial disease. For example, both HIV-1 infection and chronic obstructive pulmonary disease (COPD) impair alveolar macrophage (AM) killing of pneumococci (16, 17). Furthermore, pathogenic bacteria have evolved mechanisms to withstand microbicides, such as antioxidant systems (18). Successful pathogens such as *S. aureus* inhibit phagosomal maturation contributing to intracellular survival (19), while others that are more readily killed may escape killing in subsets of macrophages, as exemplified by survival of pneumococci in permissive CD169+ splenic macrophages in murine and porcine models (20). Several potentially AMR pathogens such as *K. pneumoniae* and *P. aeruginosa* can subvert phagosomal maturation in macrophages (21, 22). Traditional paradigms of intracellular and extracellular bacteria are blurring and the intracellular fate of the so-called extracellular bacteria (including medically important ESKAPE pathogens, *Haemophilus influenzae* and *Streptococcus pneumoniae)* is likely a major determinant of infection outcome.

When the intracellular killing capacity of resident tissue macrophages is overwhelmed, they orchestrate recruitment of neutrophils and other inflammatory cells. Murine models of clodronate-mediated AM depletion illustrate how escalating bacterial challenge shifts the role of AM from primary effectors of bacterial clearance to regulators of the inflammatory response, with neutrophils required for pathogen clearance (15, 23). The exhaustion of macrophage clearance capacity is likely also a feature of systemic infections, as evidenced for Kupffer cells in the liver and is augmented by commensal bacteria (24). This represents the transition from sub-clinical infection to clinical disease, and signs of neutrophilic inflammation are used to establish a clinical diagnosis. The inflammatory response, however, contributes to tissue injury since potent microbicides, such as reactive oxygen species (ROS), can cause tissue injury and organ dysfunction (25). Nevertheless, this inflammatory response is essential and neutrophil deficiency results in severe bacterial infection (26). Neutrophil microbicidal responses have been extensively characterized and include ROS, AMP, divalent metal iron-sequestering proteins (e.g., lactoferrin), proteases such as the serine proteases contained in azurophilic granules (e.g., cathepsin G and neutrophil elastase) and acid hydrolases in lysosomes (26). The pre-eminence of ROS as a direct microbicidal mechanism has been challenged by observations that it is the associated ionic changes in the phagosome, activating granule-associated serine proteases, that actually mediate microbicidal killing (27). Neutrophils can also release granule contents and DNA extracellular traps to kill bacteria (28).

**Figure 1:**
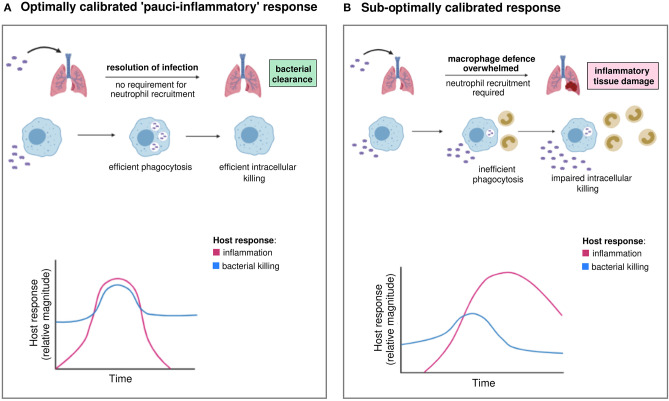
Optimal and sub-optimal inflammatory and bacterial killing trajectories during infection. **(A)** Invading extracellular bacteria are recognized and phagocytosed by macrophages, followed by intracellular killing. Pathogen clearance is optimal and achieved without the requirement for neutrophil recruitment. Inflammation is tightly controlled and resolves without causing tissue damage. We term this optimally calibrated response “*pauci-inflammatory.”* **(B)** In hosts with sub-optimally calibrated responses, there is inefficient phagocytosis and/or intracellular killing by macrophages, resulting in incomplete bacterial clearance. When macrophage defense is overwhelmed beyond a “tipping point,” neutrophil recruitment is required to control the invading pathogen. Inflammation is more prolonged and sustained by pathogen persistence and/or tissue damage. Inflammatory responses give rise to clinically recognizable features of disease, for example pneumonia. Images created using BioRender.com.

The challenge is therefore to generate an effective response that maximizes pathogen clearance and minimizes the inflammatory response, either by enhancing the macrophage response to raise the threshold for induction of neutrophilic inflammation or by ensuring the neutrophilic component achieves pathogen clearance yet limits bystander tissue injury. We term this desirable microbicidal profile a “pauci-inflammatory microbicidal response” recognizing that its characteristics include rapid induction, effective pathogen killing, and controlled recruitment of inflammatory cells when needed, but also early resolution and tightly regulated production of potentially damaging microbicidal species (Figure 1). It builds on concepts articulated by Sears and colleagues in chronic parasitic infections where the cost of the host response (immunopathology) is weighed against resistance to the pathogen (29). In the case of common “extracellular” bacterial disease, the primary cost becomes tissue injury/organ dysfunction due to the microbicidal response and chronic infection is a rare outcome. If initial microbicidal responses by phagocytes are sub-optimal, the inflammatory response is escalated with increased recruitment of neutrophils, macrophages and lymphocytes that have the potential to promote self-propagating waves of inflammation driven by release of damage-associated molecular patterns in response to tissue injury. Excessive production of cytokines, reactive species, proteases, phospholipids and eicosanoids mediate inflammatory tissue injury, induction of various cell death paradigms and ultimately loss of tissue homeostasis. These principles are well-exemplified by the development of acute respiratory distress syndrome (ARDS) (30). Organ specific injury is also associated with a systemic inflammatory response which can cause multiorgan failure (31). In addition, the generalized inflammatory response can lead to immunosuppression with impaired immune responses on subsequent pathogen challenge (32). It is therefore essential to limit these dysregulated inflammatory responses and induce a more limited inflammatory response with optimal pathogen clearance, by targeting microbicidal responses. To target potential bottlenecks in the host microbicidal response, we must identify optimal responses that promote resilience in the healthy population and patient groups in whom these fail. We need to develop assays to assess the host response and effect of therapy.

## Identifying Host Responses as Targets for Immunomodulation

A critical bottleneck in host defense involves macrophage bacterial clearance (19, 33). However, therapeutic modulation of this is impeded by limitations in our understanding of microbicidal responses in tissue macrophages, which are often inferred from neutrophils, monocytes or monocytic cell lines. Well-established microbicidal mechanisms in other phagocytes may not operate in tissue macrophages, which (excluding those in atherosclerotic plaques) lack the ability to produce the more potent halogenated ROS like hypochlorous acid (34, 35). Some microbicidal responses are more convincingly demonstrated in mice than man, for example those involving nitric oxide (NO), which may be produced at lower levels in human macrophages, although several groups have detected it following bacterial challenge (36). Effective responses likely require combinations of microbicidals. Defining these has been limited by how well *in vitro* macrophage cultures mirror tissue macrophages *in vivo*. Many tissue macrophages with low-level homeostatic turnover arise from embryonic yolk sac or fetal liver hematopoietic stem cell progenitors and are maintained by division of resident cells, e.g., AM derived from fetal liver precursors (37). Monocyte-derived macrophages (MDM) give rise to macrophages in the gut and peritoneum, populations associated with a higher turnover, but we cannot assume their microbicidal responses are identical to macrophages derived from embryonic progenitors. In addition, tissue macrophage maturation is heavily influenced by environmental cues and their transcriptional profiles are as distinct as they are from monocytes (38).

**Figure 2:**
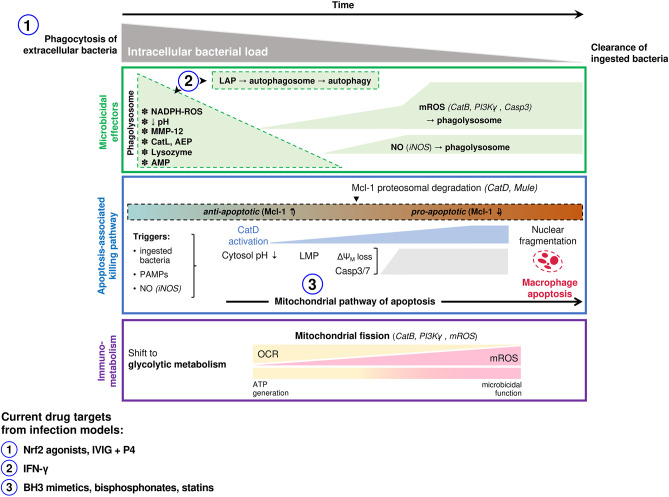
Macrophage microbicidal responses involved in successful clearance of extracellular bacterial pathogens. Macrophage responses to ingested extracellular bacteria (e.g., *S. pneumoniae, S. aureus, P. aeruginosa*) are summarized. Following phagocytosis of bacteria an initial microbicidal response occurs in the phagolysosome (top panel). Specific effectors with demonstrated microbicidal roles differs based on the ingested organism, and include NADPH derived ROS, MMP-12 (*S. aureus*), cathepsin L (*S. aureus*), asparagine endopeptidase (*P. aeruginosa*), lysozyme and antimicrobial peptides. Microbicidal species produced later that co-localize to bacteria-containing phagolysosomes include NO and mROS which have demonstrated roles in killing ingested pneumococci. A mitochondrial pathway of host-directed apoptosis is engaged in response to live ingested pneumococci, involving recognition of pneumolysin and accumulation of NO (middle panel). This has been best studied in pneumococcal models, where it allows pauci-inflammatory clearance of bacteria that have survived initial phagolysosomal killing, but may occur for other extracellular bacteria also. Immuno-metabolic changes that underpin the microbicidal function of macrophages have also been characterized well in pneumococcal models and also in some other extracellular bacterial infections (bottom panel). This involves an early shift to glycolysis and a progressive transition of mitochondrial function from ATP generation (oxidative phosphorylation) to become microbicidal organelles (mROS generation). Targets of host-directed therapeutics that have been investigated in infection studies (clinical or pre-clinical) are indicated. The number corresponding to each indicates the stage in the killing process where it acts, as indicated on the panels above. LAP, LC-3 associated phagocytosis; MMP, matrix metalloproteinase; Cat, cathepsin; AEP, asparagine endopeptidase; AMP, antimicrobial peptide; ROS, reactive oxygen species; mROS, mitochondrial ROS; NO, nitric oxide; PI3K, phosphoinositide 3-kinase; Casp, caspase; iNOS, inducible nitric oxide synthase; Mcl-1, myeloid cell leukemia-1; PAMP, pathogen-associated molecular pattern; LMP, lysosomal membrane permeabilization; **Δ**Ψ_M_, mitochondrial membrane potential; OCR, oxygen consumption rate; IVIG, intravenous immunoglobulin; IFN, interferon.

Irrespective of these limitations there are many similarities between microbicidal mechanisms of different macrophage populations. A range of primary human macrophages (including MDM and AM) and murine models demonstrate an initial phase of extensive intracellular killing, activated in the phagosome. For pathogens such as pneumococci, this is followed by a delayed phase of bacterial killing, involving apoptosis-associated killing that clears residual viable bacteria (16, 19, 33). These responses often involve combinations of microbicidals (Figure 2), for example ROS and NO, which helps subvert pathogen resistance (33). Tissue macrophages modify the phagosomal environment to inhibit bacterial survival; phagolysosomal acidification and restriction of divalent metal cations inhibits bacterial enzymes, including manganese-containing superoxide dismutase. Nevertheless, the role of these responses is more established in killing intracellular bacteria, compared to internalized extracellular bacteria (39). These defenses are complemented by AMP and proteases. Matrix metalloproteinase 12 contributes to early killing of bacteria in macrophages (40). The cathelicidin LL-37 enhances killing of bacteria including *S. aureus* in macrophages and is taken up from exogenous sources to complement ROS generation and lysosome fusion (41). AMR in *E. coli* can increase the sensitivity to AMP, suggesting host-based strategies can synergize with antimicrobials or with antimicrobial selective pressure (42). Similarly, a synthetic peptide derived from human lactoferrin synergizes with antimicrobials against a carbapenemase-producing *K. pneumoniae* (43). However, there are also examples where mutations inducing AMR may also enable resistance to AMP; modification of *K. pneumoniae* lipid A not only enables resistance to polymyxins but also β-defensins and human neutrophil peptide-1 (44). Many other AMP and proteases contribute to microbicidal responses, but the mechanism may be indirect. For example cathepsin D enhances apoptosis-associated killing by increasing proteasomal degradation of the anti-apoptotic Bcl-2 family member Mcl-1 (45).

The ability to perform lentiviral delivery of genome-scale clustered regularly interspaced short palindromic repeats (CRISPR)-associated nuclease Cas9 knock-out (GeCKO) pooled libraries to human cells allows whole genome screening with the potential to shed new light on microbicidal mechanisms (46, 47). A further potential approach is to harness comparative biology and aims to use convergent evolution of pathogens as they shift species tropism (48) or divergent evolution within species as they rapidly evolve under a host-selective pressure (49), to probe microbicidal mechanisms. Nevertheless, identifying microbicidal mechanisms as targets for immunomodulation will also require evidence that these are sub-optimally calibrated in patient groups with increased susceptibility to bacterial disease. For example, AM from patients with COPD fail to enhance mitochondrial ROS (mROS) production following bacterial challenge (16). This is important since mROS has recently emerged as a key microbicide affecting bacterial killing in the macrophage phagolysosome (33, 50). Evaluation of potential microbicidal targets will also require application of super-resolution microscopy and other advanced imaging modalities, combined with advances in probes, optics and analytics to provide temporal and spatial resolution of microbicidal generation. In the past, generation at a population level using automated systems such as flow cytometry has been assumed to be a surrogate for this but may be insufficient to allow optimal characterization. *In vivo* imaging is also a valuable adjunct and comparative medicine using large animals such as pigs, whose immune system is similar to humans, and studies in humans will aid translation in models of infection (51, 52).

## Recalibrating Microbicidal Responses in Clinical Settings

Only a few strategies to modulate the host response to bacteria have progressed to clinical trials, and specific assessment of target microbicidal responses is often lacking (Table 1). Interferon (IFN)-γ is established in the treatment of chronic granulomatous disease (CGD), a genetic disorder in which deficiency in one of the components of the nicotinamide adenine dinucleotide phosphate (NADPH) oxidase leads to increased susceptibility to a range of infections. While this is an extreme case of adjusting an immune response, it shows immunomodulation can be used to enhance microbicidal responses. Clinical trial data shows IFN-γ reduces the frequency of severe infections in CGD and it has also been investigated for multi-drug resistant tuberculosis, *Mycobacterium avium* complex and *Cryptococcus neoformans* infections (53, 68). IFN-γ enhances several microbicidal mechanisms and has been shown to correct defective *ex vivo* killing of the intracellular pathogen *Burkholderia cenocepacia* in cystic fibrosis (CF) MDM by enhancing autophagy, a regulated cellular process that enables removal and recycling of macromolecules and organelles to promote cellular homeostasis and a related cell process using autophagy machinery that leads to killing of ingested bacteria termed xenophagy (59). However, nebulized IFN-γ did not reduce bacterial density or inflammation in a clinical trial in CF (69). In critically ill adults, clinical trial data demonstrates that IFN-γ is associated with clearance of persistent bacteremia and improved cytokine profiles in the setting of sepsis-induced immunosuppression. Further investigation in clinical trials in sepsis is ongoing (70). It has also been shown to correct HLA-DR expression on monocytes in patients with sepsis which provides a useful marker of response (56). In a case report, IFN-γ enabled clearance of persistent *S. aureus* bacteremia in association with transcriptional profiles associated with a shift toward Th1/Th17 responses and antigen-specific T-regs, though the specific consequences for microbicidal responses were not examined (58). In patients with septic shock and lymphopenia, IL-7 has been shown to reverse sepsis-induced lymphopenia (55).

**Table 1:** Examples of host-directed therapies in infectious diseases from clinical and pre-clinical studies.

**Therapy**	**Level of evidence**	**Target**			**Pathogen or disease**	**Outcomes**	**References**
		**Cell type**	**Cellular pathway**	**Microbicidal response**			
IFN-γ	Clinical trial (RCT)	Neutrophil	NADPH-mediated ROS production	Phagosomal intracellular killing	Patients with chronic granulomatous disease (*n* = 128)	↓ frequency of serious infections in patients receiving IFN- γ (22 vs. 46%, *p* = 0.0006).	(53)
						No serious toxicity.	
GM-CSF	Clinical trial (RCT)	Neutrophil	RhoA GTPase pathway and actin polymerisation	Phagocytosis	Critically ill adults with ↓*ex vivo* neutrophil phagocytosis (*n* = 38)	*Ex vivo* reversal of defective neutrophil phagocytosis.	(54)
						No serious toxicity.	
IL-7	Clinical trial (RCT)	Lymphocyte	IL-7R signaling via Jak/STAT and PI3K/Akt pathways	T-cell apoptosis	Adults with septic shock and lymphopenia (*n* = 27; most commonly pneumonia or intra-abdominal infection)	↑ absolute lymphocyte count.	(55)
						↑ CD8^+^ and CD4^+^ T-cell count.	
						↑ T-cell proliferation and activation.	
						No serious toxicity	
IFN-γ	Clinical trial	Monocyte	HLA-DR expression	Monocyte activation	Critically ill adults with sepsis and ↓ monocyte HLA-DR expression (*n* = 9)	↑*ex vivo* monocyte LPS-induced TNF-α production.	(56)
						↑*ex vivo* monocyte HLA-DR expression.	
						No serious toxicity.	
Anti-PD1 mAb + IFN-γ^*^	Case report	Lymphocyte	PD-1/PDL-1 interactions	T-cell apoptosis	1 patient with invasive mucormycosis	Clinical cure.	(57)
						↑ absolute lymphocyte count.	
						↑ monocyte HLA-DR expression.	
						↑ CD8^+^ T-cell count.	
						↓ T-cell PD-1 expression.	
IFN-γ^*^	Case report	Monocytes	HLA-DR expression	Monocyte activation	1 patient with persistent *S. aureus* bacteraemia and metastatic infection	Clinical cure.	(58)
						↑ MHC-II pathway transcription.	
						↑ HLA-DR expression.	
						↑ antigen-specific T-reg cells.	
						Shift from Th2 to Th1/Th17.	
IFN-γ	Pre-*clinical*	Macrophage	p62 tagging of intracellular bacteria and autophagosome formation.	Autophagic killing of intracellular bacteria	*B. cenocepacia* (cystic fibrosis)	MDM from patients with cystic fibrosis *in vitro*:	(59)
						↑ intracellular killing	
						↓ IL-1β production	
P4 peptide + IVIG	Pre-clinical	Neutrophils and macrophages	Fc-γR	Phagocytosis	*S. pneumoniae*	Murine pneumococcal disease model:	(60)
						↑ survival	
						↑ bacterial clearance	
						↑ Fc-γR expression (neutrophils)	
						*M*urine macrophages: ↑ phagocytosis.	
P4 peptide	Pre-clinical	Neutrophils and monocytes	Phagosome	Phagocytosis and killing	*S. pneumoniae*	Neutrophils from adults with severe sepsis:	(61)
						↑ neutrophil bacterial killing	
						↑ neutrophil and monocyte ROS	
Nrf2 agonists	Pre-clinical	Macrophage	Antioxidant response (phase II detoxifying enzymes)	Phagocytosis	*S. pneumoniae, H. influenzae*	Alveolar macrophages from patients with COPD: ↑ phagocytosis.	(62)
BH3 mimetics Clodronate	Pre-clinical	Macrophage	Inhibition of anti-apoptotic BCL-2 family members or induction of apoptosis in case of clodronate	Apoptosis-associated killing	*S. pneumoniae, L. pneumophila*	Murine pneumonia models:	(33, 63)
						↑ survival	
						↑ bacterial clearance (lung)	
						↓ neutrophil recruitment	
						↑ alveolar macrophage apoptosis	
Statins	Pre-clinical	Macrophage	Cholesterol biosynthesis	Phagosomal maturation and autophagy	*M. tuberculosis*	MDM from statin-treated patients:	(64)
						↓ intracellular bacterial growth	
						Murine tuberculosis model:	
						↓ bacterial burden and lung micro-abscesses	
						Statin-treated murine BMDM:	
						↓ intracellular bacterial growth	
Statins	Pre-clinical	Macrophages	Cholesterol biosynthesis	Apoptosis-associated killing	*S. enterica* serovar Typhimurium	Statin-treated RAW 264.7 cells:	(65)
						↓ intracellular bacterial growth	
						↑ apoptosis and CatD localisation to SCV	
						Murine model (intra-peritoneal):	
						↓ bacterial burden (liver and spleen)	
Statins	Pre-clinical	Neutrophils	Cholesterol biosynthesis	NETosis Phagocytosis ROS	*S. aureus*	Statin-treated neutrophils:	(66)
						↑ extracellular killing & NETosis	
						↓ phagocytosis	
						↓ oxidative burst	
						Murine pneumonia model:	
						↑ bacterial clearance (lung)	
						↓ lung inflammation	
						↑ NETosis	
Statins	Pre-clinical	Macrophages	Cholesterol biosynthesis JNK pathway	Phagocytosis ROS Fc-γR signaling	*S. aureus*	Statin-treated MDM:	(67)
						↓ phagocytosis, ROS & intracellular killing	
						↑ Fc-γR-mediated TNF-α production	

*GM-CSF: granulocyte-macrophage colony-stimulating factor; IL: interleukin; IFN: interferon; RCT: randomised-controlled trial; ROS: reactive oxygen species; mAb: monoclonal antibody; IVIG: intravenous immunoglobulin; BMDM: bone marrow-derived macrophages; MDM: monocyte-derived macrophages; CatD: cathepsin D; SCV: Salmonella-containing vacuole; NET: neutrophil extracellular trap*.

* *Administered in addition to appropriate antimicrobials*.

GM-CSF and G-CSF enhance macrophage and neutrophil phagocytosis and microbicidal responses *in vitro* and are used to restore functional phagocyte numbers in patients receiving bone marrow-suppressive chemotherapy. GM-/G-CSF have also been investigated in patients with sepsis, with a meta-analysis suggesting a trend toward benefit (71, 72). Timing may be important with GM-CSF and it may have most efficacy when targeted to patients with low monocyte HLA-DR (73). Whilst the impact on microbicidal responses is often not studied, a recent clinical trial showed GM-CSF targeted to critically ill patients with defects in *ex vivo* neutrophil phagocytosis could ameliorate this defect and increase monocyte HLA-DR (54). Both GM-CSF and IFN-γ will, with subtle differences, contribute to macrophage activation phenotypes that promote microbicidal responses, particularly against pathogens with significant intracellular survival. Other cytokines will have similar effects (74). As with many other approaches listed, each can impact more than one cellular process directly or indirectly, affecting microbicidal responses (Table 2). For example, IFN-γ can also enhance myeloid cell recruitment in clinical trials (68).

**Table 2:** Summary of strategies of host-directed therapy.

**Strategy**	**Therapy**	**References**
↑ microbicidal activity through canonical killing mechanisms	IFN-γ	(53)
	GM-CSF	(54)
	Statins (undetermined mechanism, presumed canonical)	(64–66)
	Anti-PD1 (nivolumab)	(57)
	IL-7	(73)
	P4 peptide	(61)
↑ apoptosis-associated	BH3 mimetics	(33, 63)
killing (macrophages)	Clodronate	(33)
	Statins	(65)
↑ xenophagy	IFN-γ	(59)
	Statins	(64)
	PI3K, MAPK 5′ AMP kinases	(75)
↑ monocyte activation	IFN-γ	(56, 58)
	GM-CSF	(54, 73)
	PDL1 inhibitor	(76)
Enhancing T cell numbers to indirectly increase microbicidal responses	IL-7	(73)
↑ Phagocytosis as basis of increased microbicidal response	GM-CSF	(54)
	IVIG	(60)
	P4 peptide	(61)
	Nrf2 agonists	(62)
	Statins	(66, 67)

Other investigational approaches include the use of check-point inhibitors, such as anti-programmed cell death protein-1 (anti-PD-1) or anti-cytotoxic T-lymphocyte-associated protein-4 (CTLA-4) monoclonal antibodies (73). These inhibitors aim to reverse suppression of T-cell inflammatory responses. Nivolumab, an anti-PD-1 monoclonal antibody, is being tested in a clinical trial in sepsis, and while such therapies are anticipated to modulate the inflammatory response, they may also target microbicidal responses. For example, there is a case report of Nivolumab being used in combination with IFN-γ to successfully treat an intractable fungal infection (57). A PD-1 ligand inhibitor has also been shown to increase monocyte HLA-DR expression (76). Other immune modulating strategies that can be expected to modulate microbicidal responses include recombinant IL-7, which corrects lymphopenia and will enhance IFN-γ, and intravenous immunoglobulin (IVIG), which in addition to immunomodulation enhances pathogen clearance through phagocytosis (73). Immunomodulatory peptides have also been combined with IVIG, specifically the P4 peptide (derived from the immunomodulatory pneumococcal lipopeptide Pneumococcal surface adhesin A), resulting in increased pneumococcal clearance in mice and enhanced neutrophil and monocyte bacterial killing (60, 61).

## Repurposed Drugs to Target Microbicidal Responses In Pre-clinical Models

Studies in relevant *in vitro* and animal models, and human patient groups, can identify host microbicidal targets. But there is then a need to develop therapeutic approaches to modulate these targets. This will inevitably be constrained by cost, but this can potentially be reduced by re-purposing existing agents that are found to modify the host response of interest (75).

Critical illness can be associated with the compensatory anti-inflammatory response syndrome and temporary immunoparesis, after the initial stages of innate immune activation. This is characterized by reduced Th1 and monocyte responses, which increase the risk of nosocomial infection (77). Reducing PRR engagement and subsequent immune activation, such as through reduction in TLR activation in the early stages of illness, could potentially reverse this phenomenon and the turmeric constituent curcumin appears to down-regulate signaling through a range of TLRs (78, 79).

Phagocytosis of bacteria activates phagosomal microbicidal responses in myeloid cells (80). Although phagocytosis is not usually a rate limiting process, in conditions such as COPD macrophage phagocytosis may be reduced. This is associated with increased airway bacterial burden (62). This defect is related to cellular oxidative stress (62, 81). Nrf2 agonists are in development, which enhance the host cell's anti-oxidant host defenses, and in COPD AM can enhance phagocytosis as well as clearance of *P. aeruginosa* in mice exposed to cigarette smoke (62, 82).

Xenophagy is selective autophagy that aids clearance of intracellular pathogens such as *Mycobacterium tuberculosis* (83) and some extracellular bacteria. Of note, *Streptococcus pyogenes* subverts this process in endothelial cells (84). Activation of autophagy via inhibition of inhibitory pathways, such as class I phosphoinositide-3-kinase, mitogen-activated protein kinases or 5'-AMP-activated protein kinases, could be a tractable microbicidal strategy and drugs already under development for other indications could be re-purposed (75).

Another novel microbicidal response in macrophages and potentially other myeloid cells involves apoptosis-associated killing. BH3 mimetics enhance killing of *S. pneumoniae* and *Legionella pneumophila* in murine models through augmentation/restoration of this pathway (33, 63). Bisphosphonates also enhance macrophage apoptosis-associated killing of bacteria (33), while fluoroquinolones cause lysosomal permeabilization, sensitizing cells to this pathway (45, 85).

3-hydroxy-3-methyl-glutaryl-CoA (HMG-CoA) reductase inhibitors, termed statins, are used as cholesterol lowering medicines. Statins enhance bacterial clearance in a murine sickle cell model of pneumococcal disease. The impact was limited to the sickle cell mice with no response seen in wild type (86). One potential mechanism was downregulation of platelet-activating factor receptor required for bacterial translocation from the lung in the sickle cell mice. However, the microbicidal basis for the enhanced clearance was not established beyond the association of increased clearance with reduced sickle cell-associated inflammation. In the case of *M. tuberculosis*, statins enhance phagosomal maturation and xenophagy (64), while for *Salmonella enterica* serovar Typhimurium they enhance cathepsin D localization to phagosomes and apoptosis induction (65). Whether they also enhance these processes for extracellular pathogens is not established. They can enhance neutrophil and monocyte killing by extracellular traps (66). However, they inhibit phagocytosis and microbicidal responses in other models such as Fcγ-receptor mediated uptake of opsonized *S. aureus* (67) and reduce bacterial killing by neutrophils in a murine pneumonia model (87). Therefore, how they would be best used requires further elucidation, as reflected in contradictory findings from clinical studies. For example, a reduced risk of community-acquired *S. aureus* bacteremia (88) and reduced mortality in pneumonia were reported (89, 90) yet no reduction in mortality was observed in another pneumonia study (91) or in a study of ventilator-associated pneumonia (92).

## Challenges

Recalibrating responses will likely require a personalized medicine approach. Individual pathogens would need varying degrees of engagement of a given response. *S. aureus* inhibits apoptosis-associated killing in macrophages so might need a greater degree of enhancement, or might require an alternative approach, while for *S. pneumoniae* in which apoptosis-associated killing is already engaged, the adjustment might only need to be of a more modest extent in a subset of individuals (33). Certain responses might need engagement in select patient groups such as those with medical comorbidities that adjust the response. Alternatively these responses might not be suitable for enhancement in certain groups. For example, patients with COPD might not be amenable to enhancement of mROS production or might require reduction in high baseline levels of antioxidants to enhance this microbicidal response (16). Such personalized approaches would require validated tests to help calibrate an individual response.

Another challenge is that where responses need to be recalibrated it will be important that responses do not over shoot and result in overproduction of factors that could lead to tissue injury if there is excessive production of microbicidals or inflammatory cells (30). This is most likely to be prevented where the responses enhanced are intracellular, generated at high levels adjacent to bacteria and transient. Responses will require application of techniques to measure the individuals response through use of appropriate biomarkers or imaging modalities and would benefit from approaches that combine these measures with microdosing experiments and endomicroscopy (the application of *in vivo* microscopy applied through endoscopy to allow optical biopsy) to test the efficacy of recalibration (93).

## Conclusions

The ineluctable progression of AMR necessitates investigation of novel strategies for treating bacterial disease. Based on the observation that exposure to potentially pathogenic bacteria infrequently leads to disease, we contend that identification and exploitation of specific determinants of host defense represents a tractable alternative to antimicrobials (host-based therapy). While there are many potential aspects of the host response that represent tractable targets, including humoral factors (e.g., AMP), epithelial barrier function, and lymphoid populations, we suggest approaches that promote pauci-inflammatory macrophage and neutrophil microbicidal responses can improve outcomes. We have highlighted a number of promising *in vitro*, animal model, human and pre-clinical observations that support this viewpoint and provide a roadmap for future research.

## Author Contributions

KW, CR, and DD wrote the initial drafts of the article. JB, KD, JF, TM, AS, and SR provided critical comment and revised the document.

## Conflict of Interest

The authors declare that the research was conducted in the absence of any commercial or financial relationships that could be construed as a potential conflict of interest.
